# Distinct effects of isoflurane on basal BOLD signals in tissue/vascular microstructures in rats

**DOI:** 10.1038/srep38977

**Published:** 2016-12-15

**Authors:** Tomokazu Tsurugizawa, Yukari Takahashi, Fusao Kato

**Affiliations:** 1 Department of Neuroscience, The Jikei University School of Medicine, 3-25-8, Minato-ku, Tokyo 105-8461, Japan; 2 Neurospin, Commissariat à l’Energie Atomique et aux Energies Alternatives, Bat 145, Point Courrier 156, Gif-sur-Yvette 91191, France

## Abstract

Isoflurane is a well-known volatile anesthetic. However, it remains equivocal whether its effects on BOLD signal differ depending on the types of intracranial structures, such as capillaries and large blood vessels. We compared dose-dependent effect of isoflurane on the basal BOLD signals in distinct cerebral structures (tissue structure or large vessels) using high resolution T2*-images at 9.4 T MRI system in rat somatosensory cortex. The local field potential (LFP) in the somatosensory cortex and mean arterial pressure (MAP) were also investigated. Isoflurane induced inverted U-shaped dose-dependent change in BOLD signal in large vessels and tissue regions: BOLD signal under 2.0% and 2.5% isoflurane significantly increased from the maintenance dose (1.5%) and that under 3.0% was similar to maintenance dose. Remarkably, BOLD signal increase in tissue regions under 2.5% was significantly smaller than that in large vessels. The MAP decreased monotonically due to the dose of isoflurane and the LFP was strongly suppressed under high dose (2.5% and 3.0%). These results indicate that isoflurane-induced alteration of MAP and neuronal activity affected BOLD signal and, especially, BOLD signal in the tissue regions was more affected by the neuronal activity.

The commonly used functional MRI (fMRI) method is the blood oxygenation level-dependent (BOLD) contrast^1^. This means that, in addition to the changes in tissue oxygen consumption, those in the regional cerebral blood flow (rCBF) and/or the regional cerebral blood volume (rCBV) produce a signal increase in T2*-weighted MR images. However, various manipulations would affect the rCBF and/or rCBV as well as cerebral metabolic rate of oxygen (CMRO^2^). For example, a use of anesthetics, being necessary in most of animal fMRI studies and in some of human studies, cannot avoid changes in rCBF and thus BOLD signals even at their maintenance doses^2^,^3^,^4^,^5^.

Isoflurane is most frequently used volatile anesthetic for its ease in introduction and maintenance and the smaller risk of side effect. However, it affects the rCBF in rats^3^,^6^, monkeys^7^ and humans^8^ besides its potent neuronal suppression, complicating again the interpretation as to the origin of BOLD signal changes. For example, the enhanced sensory-evoked BOLD responses in the rat somatosensory cortex at higher isoflurane dose (2.0%) is not linearly related to the neuronal activation^9^. Furthermore, long-lasting isoflurane anesthesia results in negative BOLD responses in rat cortex^10^. Those results indicate that the interpretation of the fMRI data collected in the presence of the anesthetics should be made with care especially in the cases where the basal BOLD signal is already elevated due to the vasodilation by the drugs^9^,^11^.

Indeed, a recent study using ultra-high magnetic field (UHF) MRI has shown that temporal patterns of the BOLD signals in response to physiological stimulation are different between capillary and large vessels (artery and vein)^12^. Therefore, we hypothesized that isoflurane effects on BOLD signals were different between those from capillaries and large vessels. To establish basis to understand such potential effects, we investigated the dose-dependent effect of isoflurane on the basal BOLD signals separately in tissue regions (including capillary) and large vascular structures (including artery and vein) in rat somatosensory cortex by means of high resolution imaging at UHF MRI system. Furthermore, we compared these basal BOLD changes with neuronal activity (local field potential, LFP) and mean arterial pressure (MAP).

## Results

### Dose dependent effect of isoflurane on BOLD signal was vascular structure-dependent

**Figure 1:**
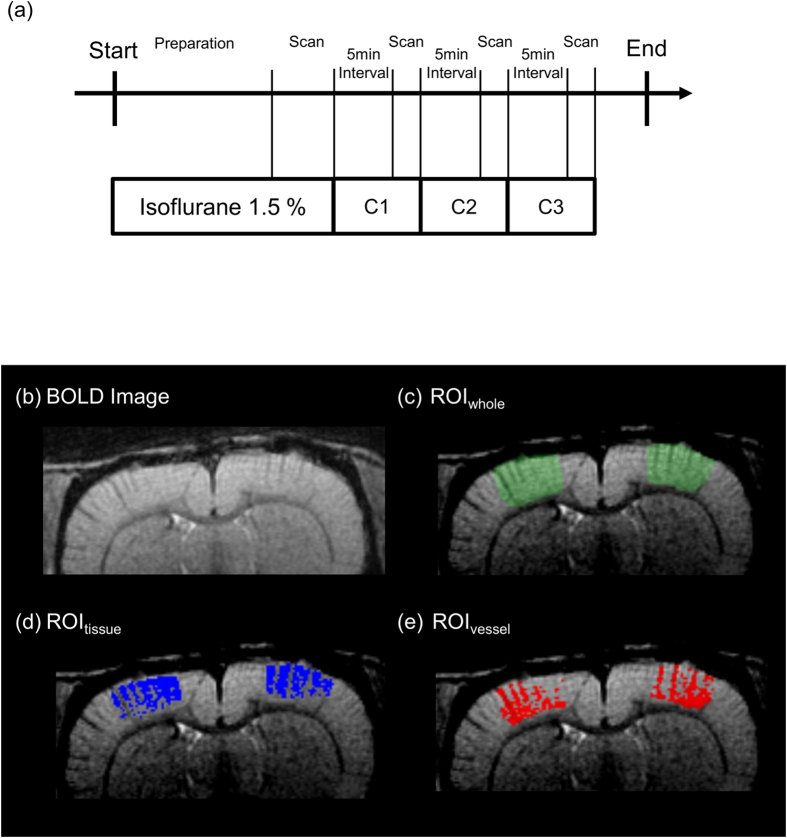
Experimental paradigm and representative ROIs. (**a**) MRI Scanning paradigm. During the MRI setup (preparation period) and first scan of BOLD image, the rats were anesthetized with 1.5% isoflurane. Then, isoflurane concentration (C1–C3) was changed randomly (2.0%, 2.5% or 3.0%). After the change to anesthetic condition, the scanning was done after 5 min of interval. (**b**) Raw T2* BOLD image. (**c**) ROI of somatosensory cortex (ROI_whole_, green), (**d**) ROI of tissue regions (ROI_tissue_, blue), and (**e**) ROI of large vessels (ROI_vessel_, red) in somatosensory cortex with high contrast T2*-image.

BOLD images were acquired in the same rats under 1.5% (maintenance concentration), 2.0%, 2.5% and 3.0% (supra-anesthetic doses) isoflurane (Fig. 1a). Then, regions of interests (ROIs) of tissue (ROI_tissue_) and large vessels (ROI_vessel_) were automatically discriminated from ROI of the somatosensory cortex (ROI_whole_) by means of Otsu’s analysis method (Fig. 1b–e). BOLD signals in the somatosensory cortex (BOLD_whole_), in the tissue (BOLD_tissue_) and in the vessels (BOLD_vessel_) were compared.

**Figure 2:**
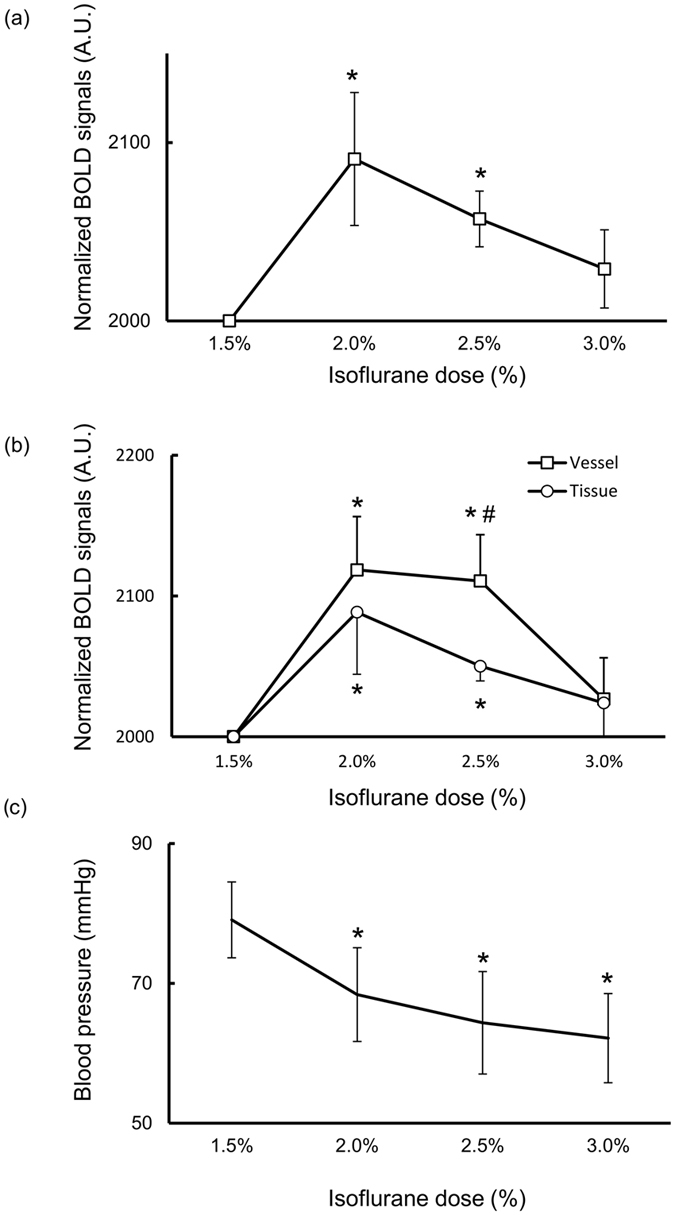
Isoflurane-dose dependency of BOLD signal and MAP. (**a,b**) BOLD signal intensity (**a**) in the somatosensory cortex (BOLD_whole_), (**b**) in the tissue regions (BOLD_tissue_) or large vessels (BOLD_vessel_) at each isoflurane concentration. Vertical axis is the BOLD signal intensity that was normalized to 2000 under 1.5% isoflurane (arbitrary unit (A.U.)) *P < 0.05, compared to 1.5% by paired t-test. ^#^*P* < 0.05, compared to BOLD signal in tissue regions at each concentration by t-test following two-way repeated ANOVA. (**c**) MAP at each isoflurane concentration. *P < 0.05, compared to 1.5% by paired t-test. Data are expressed as mean ± SEM.

The isoflurane dose-dependency of BOLD signals in the somatosensory cortex (BOLD_whole_) is shown in Fig. 2a. The BOLD_whole_ intensity shows the inverted U-shaped dose-dependency of isoflurane: BOLD_whole_ under 2.0% and 2.5% isoflurane significantly increased from the maintenance dose (1.5%) and BOLD_whole_ under 3.0% was similar to maintenance dose (2091 ± 38 under 2.0%; *P* = 0.035, 2057 ± 16 under 2.5%, *P* = 0.011; 2029 ± 22 under 3.0%, *P* = 0.127 respectively). Then normalized signal changes in BOLD signals in the tissue (BOLD_tissue_) and those in the vessels (BOLD_vessel_) in the somatosensory cortex were compared at different isoflurane concentrations (Fig. 2b). BOLD_tissue_ and BOLD_vessel_ showed the inverted U-shaped dose-dependency like BOLD_whole_. The BOLD signal intensity at 2.0 and 2.5% became significantly larger than that under 1.5% in both BOLD_tissue_ (2088 ± 44 under 2.0%, *P* = 0.048; 2050 ± 11 under 2.5%, *P* = 0.004; 2023 ± 30 under 3.0%, *P* = 0.240, respectively) and BOLD_vessel_ (2119 ± 38 under 2.0%, *P* = 0.018; 2111 ± 33 under 2.5%, *P* = 0.014; 2027 ± 29 under 3.0%, *P* = 0.207, respectively). Remarkably, BOLD_vessel_ under 2.5% was significantly larger than BOLD_tissue_ (*P* = 0.043).There was no significant difference of voxel number among all dosages of isoflurane (Table 1).

### Dose dependent effect of isoflurane on MAP

The MAP significantly decreased under 2.0% to 3.0% (68 ± 7 mmHg under 2.0%, *P* = 0.012; 64 ± 7 mmHg under 2.5%, *P* = 0.006; 62 ± 6 mmHg under 3.0%, *P* = 0.016, respectively) compared with 1.5% (79 ± 5 mmHg) (Fig. 2c).

### Dose dependent effect of isoflurane on LFP

**Figure 3:**
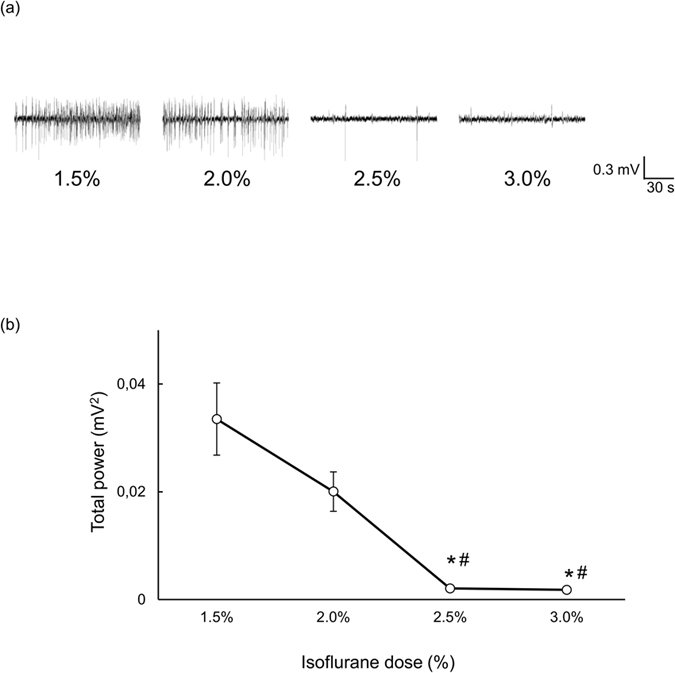
Isoflurane-dose dependency of LFP in the somatosensory cortex. (**a**) Representative LFP signals. (**b**) Averaged LFP power under each dosage of isoflurane. *P < 0.05, compared to 1.5% and ^#^*P* < 0.05, compared to 2.0% by Tukey-Kramer multiple comparisons. Data are expressed as mean ± SEM.

Dose dependency of LFP is shown in Fig. 3. LFP in representative rat shows the significant decrease of LFP under 2.5% and 3.0% isoflurane (Fig. 3a). The average of LFP power under 2.0% decreased but not significantly compared to 1.5% (0.033 ± 0.006 mV^2^ under 1.5%; 0.020 ± 0.003 mV^2^ under 2.0%, *P* = 0.13). Then, LFP power under 2.5% and 3.0% further significantly decreased compared to those under 1.5% and 2.0% (0.0020 ± 0.0004 mV^2^ under 2.5%, *P* = 0.002 vs 1.5% and *P* = 0.042 vs 2.0%; 0.0018 ± 0.0003 mV^2^ under 3.0%, *P* = 0.017 vs 1.5% and *P* = 0.039 vs 2.0%, respectively) (Fig. 3b).

## Discussion

The results presented here with a use of non-invasive high-resolution imaging with UHF MRI successfully demonstrate that basal BOLD signal in tissue structure was less influenced by isoflurane than large vessels. Further, the BOLD signal changes differed from MAP changes because BOLD signal changes are not monotonic (Fig. 2). This discrepancy between BOLD signals and MAP could be explained by stronger suppression of neuronal activity under 2.5% and 3.0% isoflurane than 1.5% and 2.0% isoflurane. The basal BOLD signal is a key factor for “BOLD response” to physiological stimulation (e.g., somatosensory stimulation and visual stimulation) and “resting state functional connectivity”. If basal BOLD signals are altered by anesthetics or vasoactive drugs, such as vasodilator or vasoconstrictor, observed BOLD response would be also altered compared to normal state^11^. Therefore, it is important to investigate the effect of vasoactive drugs on basal BOLD signals.

One of the important novel findings of this study is that the effects of isoflurane on the basal level of BOLD signals depend on the type of blood vessels in the somatosensory cortex. Such vascular-structure dependent BOLD responses are in agreement with the previous UHF MRI study in the Whisker-Barrel cortex of medetomidine-sedated rats^12^. Also, region-dependent differences in the BOLD responses have been demonstrated using UHF MRI in the auditory cortex of cats^13^ and visual cortex of human subjects^14^. These studies, including ours, took advantage of UHF MRI that can provide improved spatial resolution and T2*-contrast through decreased signal to noise ratio (SNR) and shorter T2*-values. Therefore, on the basis of the present results, it is proposed that 1) high resolution fMRI analysis is the key for distinguishing BOLD signal components in the vascular structure and 2) anesthetics frequently used in fMRI studies would potentially affect the BOLD signals in a type-, dose-, and the target structures-dependent manners.

In the present study, because of the thickness of the images (400 μm), there would be the partial volume effect: a pixel contains both tissue and large vessels. This might have resulted in a blurred boundary between vessels and tissues. We used Otsu’s thresholding method, which was widely used and was robust to this problem^15^,^16^, and we could successfully classify the large vessels and tissues. However, for further accurate analysis, ultra-higher resolution T2*-images that are powerful enough to observe only large vessel or tissue region in a pixel should be performed in the future.

Previous studies have revealed that isoflurane dose-dependently alters the rCBF, rCBV and CMRO_2_ in the brain. Higher dose (1–2 minimum alveolar concentration (MAC)) of isoflurane not only increases the rCBF and rCBV^17^,^18^ but also decreases the CMRO_2_ in the cerebral cortex^19^ dose-dependently. The MAP is also decreased by isoflurane dose-dependently, but the partial pressure of oxygen (pO_2_), partial pressure of carbon dioxide (pCO_2_) and pH are constant^17^,^20^, consistent with our result. Therefore, from the view of vasoactive effects of isoflurane with biophysical BOLD model^21^, basal BOLD signals seem to increase monotonically due to isoflurane dose; however, highest signal increase was observed under 2.0% and then it decreased from the peak under 2.5% and 3.0% (Fig. 2). It is difficult to explain this phenomenon only with “vasoactive” effect of isoflurane.

Another factor to regulate the BOLD signals is the interaction among neurons, astrocytes and blood vessels, called as neurovascular coupling^22^. BOLD signal is positively correlated with the neuronal activity through the neurovascular coupling. The isoflurane has potent effects on inhibitory and excitatory synaptic transmission^23^,^24^,^25^ and postsynaptic excitability^26^,^27^,^28^, which comprise the core effects underlying their “anesthetic” pharmacology. The isoflurane is also known to suppress astrocyte activity even with maintenance dose. The 1.2% and 1.5% isoflurane suppresses the astrocyte activity, but this range of isoflurane does not suppress the neuronal activity^29^. Our results also show that synaptic activity was significantly suppressed from 2.5% (Fig. 3). Together, the BOLD signal increase under 1.5% and 2.0% could be mainly due to the vasodilation effects of isoflurane with remaining neuronal activity, but BOLD signal decrease under 2.5% and 3.0% compared to 2.0% could be due to the mixture of vasodilation and strong suppression of neuronal activity.

Remarkably, BOLD signal changes in tissue regions under 2.5% were smaller than those in large vessels. Together with above discussion, this indicates that BOLD signals in capillaries are more affected by neuronal activity than large vessels. Importantly, CMRO_2_, rCBF and rCBV changes coupled to neuronal activity are different in the vessel type. For instance, when neuronal activity increases, the artery increases the rCBV more than capillary, but oxygen saturation of hemoglobin extremely increases in the capillary^30^. Furthermore, neurovascular structure is different among the large vessels and capillary. Capillary receives more direct regulation from neurons and astrocytes than artery and the vein^31^.

### Conclusion

In conclusion, high resolution imaging in UHF MRI is a promising tool for investigating the functional responses by distinguishing distinct microstructures of rat brain. Therefore, UHF MRI would play an important role in future preclinical animal study. Because anesthesia is essential for animal experiments to suppress motion artifacts and to reduce stress during the painful, unpleasant stimulation, it is important to clarify the effect of anesthetics on the BOLD signals differentially in the neuronal and vascular structures, such as the arteries, veins and capillaries, which is at this moment only possible with UHF MRI.

## Methods

### Animal preparation

The 16 adult male Wistar rats (8–12 weeks, 200–300 g) were assigned as following: n = 8 for MRI; n = 4 for LFP; n = 5 for MAP. All experimental protocol received approval and were carried out in accordance with the approved guidelines from the Institutional Animal Care and Use Committee of The Jikei University and conformed to the Guidelines for Proper Conduct of Animal Experiments of the Science Council of Japan (2006) and the Comité d’Ethique en Expérimentation Animale in France. The animals were housed individually in cages under controlled temperature conditions (22 ± 2 °C) with a 12 h:12 h light-dark cycle and free access to food and water.

An intubation was made for mechanical ventilation to ensure normoxic breathing during the scanning under 1.5% isoflurane (in air) followed by an initial induction of anesthesia with 3% isoflurane lasting within 1 min. The intratracheal cannula was connected to a mechanical ventilator (SAR-830 Ventilator, CWE Inc., CA) and ventilation was made with following parameters throughout the experiments: respiratory rate = 50/min; inspiratory time: expiratory time = 1; tidal volume = 1.7 ml. The pO_2_, pCO_2_ and pH have been confirmed to be kept in normoxic and normocapnic ranges throughout the experiment, and there was no significant difference under 1.5–3.0% isoflurane^9^.

The body temperature was maintained at 36.5–37 °C using an MR-compatible circulating water heating system (CW-05G, JEIO TECH CO., LTD., Seoul, Korea). The ventilation-controlled the waveform and rate of the respiration (50/min) and body temperature were continuously monitored throughout the experiments using an MR-compatible monitoring system (Model 1025, SA Instruments, Stony Brook, NY).

### FMRI procedure

The MRI experiments were performed using a 9.4 T horizontal bore MRI scanner (BioSpec 94/20 USR, Bruker, Ettlingen, Germany) equipped with a BGA12S gradient system. A volume coil (Bruker, Ettlingen, Germany) was used for transmitting the signal and a 4-channel array coil (Bruker, Ettlingen, Germany) was used for reception of the signal. Following scout scans and magnetic field homogeneity optimization (MAPSHIM), we obtained gradient-echo BOLD images using a T2*-weighted echo planer imaging sequence with the following parameters: time of repetition = 1,500 ms, echo time = 25 ms, segmentation = 2, field of view = 21 mm × 21 mm, acquisition matrix = 210 × 210, slice thickness = 0.4 mm, (spatial resolution = 100 × 100 × 400 μm), slice gap = 1.0 mm, slice number = 3 and number of average = 20. The positions of the slices were determined using sagittal imaging and were between −3.0 and +1.0 mm from the bregma.

### Drug effect evaluation of isoflurane

The animals were first anesthetized with 1.5% isoflurane for the fMRI setup and field homogeneity correction. The rats were kept anesthetized with 1.5% isoflurane (defined as “maintenance concentration” which was almost equivalent to its MAC, 1.4%^32^). BOLD images were acquired in the same rat under 1.5% (maintenance concentration), 2.0%, 2.5% and 3.0% (supra-anesthetic doses) isoflurane. Scanning was started 5 min after changing anesthetic concentration. The sequence of isoflurane dose was set randomly (C1–C3 in Fig. 1a). The time-course of experiment procedure is shown in Fig. 1a.

### LFP measurement

The electrophysiological recording was performed separately from MRI bore as previously described^9^. The animals, first anesthetized with 1.5% isoflurane, were placed in a stereotaxic frame (David Kopf instrument, CA), and a hole centered at 4.0 mm lateral, 0.8 mm anterior from the Bregma was drilled on the left or right side of the skull. The electrode tip (<1.0 MΩ, a 1 μm tip and 0.127-mm shaft diameter, Alpha Omega Engineering, Nazareth, Israel) was positioned at a depth of 1.8–2.3 mm from the cortical surface. LFP signals were acquired at 1 kHz sampling rate using dedicated data acquisition software (Power Lab, AD Instruments, Dunedin, New Zealand). The reference electrode was positioned on the scalp.

The experimental protocol was same as fMRI (Fig. 1). After 5 min from the change of dose of isoflurane, the LFP was recorded for 2 min. Then, averaged total power of LFP for 2 min was calculated. We confirmed that LFP signal stabilized after 5 min from the change of isoflurane dose.

### MAP measurement and blood component

The measurement of MAP was performed separately from fMRI experiment. The catheters were surgically placed into tail artery and MAP was measured by means of MR-compatible monitoring system (Model 1025, SA Instruments, Stony Brook, NY). The experimental protocol was same as fMRI (Fig. 1). After 5 min from change of dose of isoflurane, the MAP during 2 min was averaged. We confirmed that MAP stabilized after 5 min from the change of isoflurane dose.

### Data analysis

#### Changes in mean BOLD signals within identified ROI

To compare the changes by anesthetic drugs between different structures, the normalized intensity from defined ROIs were calculated as follows:

First, ROI in the somatosensory cortex (ROI_whole_) was defined based on the high resolution images and rat brain atlas^33^ (Fig. 1b and c). Same ROI_whole_ was used in the same rat under all doses of isoflurane. Then, threshold to separate the ROI of the large blood vessels (ROI_vessel_) and ROI of tissue (ROI_tissue_) was automatically identified according to discriminant analysis method (Otsu’s method)^34^. The typical histogram in the ROI_whole_ has two classes: the class 1 at lower intensity corresponds to large blood vessels and the classes 2 at higher intensity corresponds to tissue regions. The Otsu’s method determines the optimum threshold for dividing the histogram into two classes (class 1 and class 2) to maximize the ratio of variance of inter-class and intra-class (F).


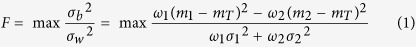


where σ_b_^2^ and σ_w_^2^ correspond to the variance of intra-class and inter-class respectively. The ω_1_ and ω_2_ are the number of voxels for class 1 and class 2 respectively. The m_1_, m_2_ and m_T_ are the averaged signal intensity for class 1, class 2 and for all voxels within the ROI_whole_ respectively. The class 1 was classified as ROI_vessel_ and the class 2 was classified as ROI_tissue_ (Fig. 1d and e). The ROI_vessel_ and ROI_tissue_ were calculated at each dose of isoflurane because the contrast of vessels might have possibly changed by vasodilation effect of isoflurane. The mean BOLD signals for each of these structures were defined as BOLD_vessel_ and BOLD_tissue_. The BOLD_vessel_ and BOLD_tissue_ intensity were normalized so that those under maintenance dose were 2000. The volumes of the large blood vessels and tissue regions were calculated by counting the number of voxels of ROI_tissue_ and ROI_vessel_. The numbers of the pixels of ROI_tissue_ and ROI_vessel_ were normalized so that those under maintenance dose were 100.

### Statistical analysis

The significance of BOLD signal intensity, MAP and LFP under supra-concentration of isoflurane (2.0–3.0%) compared with maintenance dose (1.5%) was assessed via paired t-test. The significance of BOLD signal intensity between ROI_vessel_ and ROI_tissue_ was assessed via t-test following repeated measures ANOVA.

## Additional Information

**How to cite this article**: Tsurugizawa, T. *et al*. Distinct effects of isoflurane on basal BOLD signals in tissue/vascular microstructures in rats. *Sci. Rep.* **6**, 38977; doi: 10.1038/srep38977 (2016).

**Publisher's note:** Springer Nature remains neutral with regard to jurisdictional claims in published maps and institutional affiliations.

## Figures and Tables

**Table 1:** Normalized voxel number of tissue regions and large blood vessels in somatosensory cortex.

**Tissue**			
1.5%	2.0%	2.5%	3.0%
100	96 ± 6	101 ± 5	99 ± 6
**Large vessels**			
1.5%	2.0%	2.5%	3.0%
100	112 ± 12	104 ± 10	108 ± 12

Data are expressed as mean ± SEM.
